# New EU projects delivering human microbiome applications

**DOI:** 10.2144/fsoa-2020-0028

**Published:** 2020-03-30

**Authors:** Dirk Hadrich

**Affiliations:** 1 European Commission, Healthy Lives Unit, People Directorate, Directorate-General Research & Innovation, Brussels, Belgium

**Keywords:** EU Projects, health research, international collaboration, microbiome, multidisciplinarity

## Abstract

Over recent years, the number of studies and projects showing correlations between the human microbiome and diseases has increased enormously. The potential of the human microbiome for healthcare is big. However, many confounders have been discovered and the relevant disease mechanisms and the causalities of these diseases still require more efforts and better understanding. The EU called for project proposals exploiting the application potential of human microbiome research and finally decided to fund the three top excellent new H2020 projects ONCOBIOME, MICROB-PREDICT and GEMMA. They get a total EU funding of €44 M and started in January 2019 with the aim to deliver high impact through validated clinical tools that are helpful for end-users.

## Lay abstract

Over recent years, many studies and projects have shown that the human microbiome is very important for health and diseases. The potential of the human microbiome for improving healthcare is big. However, many aspects, still require more research and better understanding. The EU called for research proposals developing useful applications and finally decided to fund the three top excellent new projects ONCOBIOME, MICROB-PREDICT and GEMMA. They get a total EU funding of € 44 M and started in January 2019 with the aim to deliver high impact for our society through validated clinical tools that are helpful for patients and doctors.

Over recent years, we have seen the trend that more and more attention has been given to the health of our gut microbiome [1]. Modern high-throughput measurement technologies have given us powerful tools to measure the properties of the cells and microbes, and they are constantly producing masses of new omics data. It turned out that gut microbiome can be associated with a very long and growing list of diseases. The EU has invested in a large variety of microbiome knowledge building projects in the past [2]. There are indications that our gut microbiome is playing to daily rhythms with us via immune effectors and that specific metabolites and proteins coming from microorganisms shape the responses of the host immune system [3]. These processes could consequently be triggers for metabolic dysfunction and human behavior [4]. Some studies also found strong predictive power of the human microbiome for diseases and death likelihood within the next 15 years [5]. Many different products are already on the market or under development to increase sports performance, sleeping quality, longevity, to improve diet, skin appearance or to influence lifestyle [6]. Yes, we live closely together with our microbes but these effects as well as diseases are very complex and the knowledge on the role of potential confounders is often insufficient so that molecular mechanisms and causalities remain in the dark [7].

## Three new top excellent EU projects are launched

Europe’s financial research and innovation instrument Horizon 2020 aims for more breakthroughs and discoveries by taking excellent ideas from the lab to the market [8]. This objective should help in boosting economic growth, creating jobs and tackling societal challenges. With scientific collaboration, we can take hurdles and facilitate the delivery of innovation.

In research on the human microbiome the challenges are to identify causalities and mechanisms in order to accelerate the translation of this knowledge into concrete tools [9,10].

The European Commission’s health work program for 2018 called for proposals to exploit the application potential of human microbiome for personalized healthcare prediction and prevention [11]. The purpose was to accelerate the translation of data and knowledge, to define balanced healthy conditions and to predict and prevent diseases through the development of personalized approaches and clinical tools. Sharing, comparing and exploiting of big biomedical data bears incredible opportunities for health research. Proposals were expected to achieve the understanding of states of health and diseases, to integrate and use high quality microbiome, metabolome and other -omics data. They should further combine and expand these data with many other disease-oriented functional data, endogenous and exogenous factors, lifestyle information, ageing data, dietary data, environmental data, etc.

27 project proposals were received before the submission deadline of 18 April 2018. Independent external experts had been appointed to assess and evaluate all proposals in detail. Finally, it was decided to fund the three project proposals that were most excellent concerning science, impact and implementation (Table 1):

**Table 1.** Three new EU microbiome projects.

Project	EU fund	Duration	Partners	Coordinator	Ref.
ONCOBIOME	€ 14.99 M	1 January 2019–31 December 2023 (60 months)	17	Laurence Zitvogel	[12]
MICROB-PREDICT	€ 15.00 M	1 January 2019 – 31 March 2025 (75 months)	22	Jonel Trebicka	[13]
GEMMA	€ 14.22 M	1 January 2019 – 31 December 2023 (60 months)	16	Alessio Fasano	[14]

ONCOBIOME [12] aims to discover the functional role of the microbiome in the tumorigenesis of four types of cancer: breast cancer, colon cancer, melanoma and lung cancer. Pathophysiological interconnections between microbes, metabolism and immunity should be validated from the project’s work. The consortium wants to create large cohorts enrolling >9000 cancer patients across ten countries. From big data on these patients specific Gut OncoMicrobiome Signatures should be validated concerning their association with the cancer occurrence, prognosis, progression and therapeutic response. Many microbiome functionalities have already been proposed but their relevance and mechanism in terms of host metabolism, immunity and oncogenesis need to be clarified [15,16]. Clinical parameters, diets, lifestyle, genomics, immunoomics, metabolomics and medications should be simultaneously analyzed and integrated with the discovered signatures and combined with economical and psycho-sociological evidence. The work of ONCOBIOME is expected to establish a very big, advanced and most useful microbiome database for cancer patients with a cutting-edge future screening strategy based on risks and personalized stool analysis.

MICROB-PREDICT [13] focuses on the link between the microbiome and the decompensation of liver cirrhosis and progression toward acute-on-chronic liver failure. There are substantial differences among patients in the disease progression and they are not at all clearly understood [17,18]. The consortium wants to deal with existing and new big biomedical data from >10,000 patients with the intention to discover the mechanisms behind the progression and decompensation of the disease and to clarify the role of confounders. Major taxonomic and functional microbial traits and their interactions should be identified. The gut microbiome, the epithelial barrier, microbial translocation and systemic inflammation have been identified as crucial triggers so that longitudinal microbiome characterization in stool, blood, gastrointestinal biopsies and saliva should be done. In parallel, other predisposing factors, genetics, exogenous factors, infections, lifestyle, diets and drugs should be longitudinally analyzed in depth and integrated. This approach should help to clearly understand all interactions, to identify functional microbial traits and causalities and to properly control confounders.

GEMMA [14] will study the development of Autism Spectrum Disorders and how they are influenced by the genome, environment, metabolome, immune system and other factors. The risk factors leading to the onset of the disorders in some children are multifactorial and not yet clearly understood [19,20]. The consortium wants to monitor a child’s development before and after disease onset, undertake regular autism assessments and deliver early diagnosis and interventions. Leading research groups in Europe and the USA are connected and collaborate in this project. They generate a unique biobank of >16,000 blood, stool, urine and saliva samples with the intention to find validated biomarkers going beyond behavioral evaluations. Causative mechanisms, functionalities and interactions between the gut microbiome, the neuroinflammation, the intestinal barrier and immune responses should be discovered. The innovative approach is using prospective patient samples, multiomics analyses, artificial intelligence, mathematical models and preclinical studies on humanized mouse models with transplanted stools from patients.

## Delivering impacts & tools

ONCOBIOME has the ambitious goal of establishing a big, unique and high-quality database from its cancer and vaccine clinical trials including chemo- and immune-therapies. An unprecedented Cancer Microbiota Atlas containing thousands of cancers should become available for the medical research community. Through machine learning techniques all data should be used to identify cancer risk factors related to microbiome specifications, lifestyle and diet. We can expect that national screening programs would need to be reorganized and enhanced to personalized risk-based stool screening for cancer. Ultimately, companion tests should be designed on the basis of the integrated signatures and advanced knowledge on microbiome immunologic and metabolic functions to predict cancer occurrence and progression. The consortium should deliver innovative diagnosis tools and GMP diagnosis kits that are specially tailored to the level of immune fitness and have high sensitivity and high specificity for at least two indications. We can be confident that novel anticancer pro and prebiotics based on dietary components or metabolites will become available on the market as prophylactic and cost-effective products.

MICROB-PREDICT should result in advanced stratification of cirrhotic patients through multifactorial assessment including microbiome analytics. Improved clinical decision-making, fewer hospitalizations of low-risk patients, better prioritization of liver transplantation, effective control of confounders and reduced healthcare costs can be expected. These intelligent and personalized approaches should help to define healthy conditions, to prevent the disease and to reduce the mortality. The consortium should identify functionalities, validate biomarkers and translate their discoveries into three new robust microbiome-based tests that are useful for patients. Ultimately, the consortium should deliver an easy-to-use and cost-effective point-of-care tool for end-users. This tool should contain microbiome-based nanobiosensors, it should be connectable to smartphones and it should allow deeper monitoring of therapeutic interventions. We can also expect that the consortium finds novel treatments that modify the microbiome, crucial mechanisms in the host and host co-factors. The European Liver Patients’ Association has an important communicative role in this project because European Liver Patients’ Association will ensure that their research results will be brought to patients, their families and to policy makers.

GEMMA’s clinical study will take blood, urine, stool and saliva samples from infants every 6 months during the first crucial 36 months of their life. In addition, they will record data on infections, antibiotics use, diets, stress and so on. This cutting-edge, prospective approach is looking at the very early disease stages at different time points and not only on the final clinical end point of neuro-inflammation in autism. The project should produce an open data repository and a multiomics platform that analyzes and integrates multiomics data. All produced and collected data should show us highly discriminative biomarkers for the onset of autism. On this basis, the consortium should run interventional tests to modulate the gut microbiome, to re-establish immune homeostasis and to deliver effective nutritional pre, pro or symbiotic formulations that prevent or change Autism Spectrum Disorders. The project will generate awareness of the burden of Autism Spectrum Disorders for the society, care providers and public authorities. Sound evidence from GEMMA should have durable impact on the food, nutrition and pharmaceutical industry to bring products on the market that are beneficial for children.

## Building bridges for our future

The three new EU projects ONCOBIOME, MICROB-PREDICT and GEMMA are ground-breaking since they accelerate the translation of data and knowledge to predict and prevent diseases through the development and validation of personalized approaches and novel valuable clinical tools. Delivering these tools enhances microbiome science from association studies to proven causative and mechanistic evidence. Intensive multidisciplinary collaborations between scientists across various sectors triggered this evolution of microbiome science from traditional metagenomic studies. The multidisciplinary approach allows a high-grade combination, comparison and exploitation of biomedical information and knowledge which may not be possible through any other route. In this way, more resources are mobilized to redefine problems and challenges beyond their normal boundaries, which finally helps us to understand complex diseases and facilitates the delivery of solutions.

This multidisciplinary approach can be further enhanced to the next level through wide international collaboration at global level because such a networking reduces the total research costs and makes the use of financial and human resources more efficient. The European Commission decided to encourage this working together process through funding the topic SC1-HCO-17-2020 that aims at ‘Coordinating and supporting research on the human microbiome in Europe and beyond’ [21]. During the next months, a project should be launched to deliver more meaningful results from collaborative synergistic collection of microbiome data from different directions under agreed methods, standards and procedures. International agreements are here required to get efficient harmonization, increased data comparability and definitive references of healthy microbiomes. Ultimately, this future cocreation process should be extended to knowledge exchange and engagement of citizens with political stakeholders. This systemic policy change of wider engagement is increasingly part of modern public administration and a way to increase the impact of public policies. Many governments have committed to higher transparency, accountability and citizen participation because this is a success factor of their policies. This wide engagement approach addresses the pressing health challenges of our society in the best way.

**Executive summary**

- The gut microbiome can be associated with a very long and growing list of diseases. The host immune system, specific metabolites and many other confounders are not yet well understood so that molecular mechanisms and causalities remain in the dark.
- Sharing, comparing and exploiting of big biomedical data bears incredible opportunities for health research. The European Commission’s health work program for 2018 aimed for research breakthrough to accelerate the translation of microbiome data and knowledge and to find useful applications to predict and prevent health.
- EU finally decided to fund these three top excellent projects:
  - ONCOBIOME is expected to establish a very big, advanced and most useful microbiome database for cancer patients with the aim to deliver innovative diagnosis tools.
  - MICROB-PREDICT should result in advanced stratification of cirrhotic patients through multifactorial assessment with the aim to deliver an easy-to-use and cost-effective microbiome-based point-of-care tool for end-users.
  - GEMMA should produce an open data repository and a multiomics platform that can analyze and integrate multiomics data with the aim to deliver effective nutritional pre, pro or symbiotic formulations that prevent or change Autism Spectrum Disorders.
- Delivering these novel tools enhances microbiome science from association studies to proven causative and mechanistic evidence. The multidisciplinary approaches allow a high-grade combination and exploitation of biomedical information and knowledge.
- The EU aims to further enhance microbiome science through wide international collaboration at the global level. During the next months, another project should be launched to get collaborative comparison of microbiome data under agreed methods, standards and procedures.
